# Antimicrobial activity of hemodialysis catheter lock solutions in relation to other compounds with antiseptic properties

**DOI:** 10.1371/journal.pone.0258148

**Published:** 2021-10-07

**Authors:** Elżbieta Piątkowska, Justyna Paleczny, Karolina Dydak, Krzysztof Letachowicz

**Affiliations:** 1 Department of Pharmaceutical Microbiology and Parasitology, Medical University of Wroclaw, Wroclaw, Poland; 2 Department of Nephrology and Transplantation Medicine, Medical University of Wroclaw, Wroclaw, Poland; Universidad Autonoma de Chihuahua, MEXICO

## Abstract

Proper protection of vascular access after haemodialysis is one of the key measures for the prevention of catheter-related infections. Various substances with bactericidal and anticoagulant properties are used to fill catheters, but due to the unsatisfactory clinical effects and occurrence of adverse reactions, the search for new substances is still ongoing. In the present paper, we compared the *in vitro* antimicrobial activity of solutions used for tunnelled catheter locking (taurolidine, trisodium citrate) and solutions of substances that could potentially be used for this purpose (sodium bicarbonate, polyhexanide-betaine). The studies have been conducted on bacteria that most commonly cause catheter-related infections. The values of both minimum inhibitory concentration and minimum biofilm eradication concentration of the substances were determined. The ability of the tested substances to eradicate biofilm from the dialysis catheter surface was also evaluated. The results showed that polyhexanide-betaine inhibited the growth of all microbes comparably to taurolidine, even after ≥ 32-fold dilution. The activity of trisodium citrate and sodium bicarbonate was significantly lower. Polyhexanide exhibited the highest activity in the eradication of bacterial biofilm on polystyrene plates. The biofilm formed on a polyurethane dialysis catheter was resistant to complete eradication by the test substances. Polyhexanide-betaine and taurolidine showed the highest activity. Inhibition of bacterial growth regardless of species was observed not only at the highest concentration of these compounds but also after dilution 32–128x (taurolidine) and 32–1024x (polyhexanide-betaine). Therefore, it can be assumed that taurolidine application as a locking solution prevents catheter colonization and systemic infection development. Taurolidine displays high antimicrobial efficacy against Gram-positive cocci as well as Gram-negative bacilli. On the contrary, the lowest antibacterial effect displayed product contained sodium bicarbonate. The inhibitions of bacterial growth were not satisfactory to consider it as a substance for colonization prevention. Polyhexanidine-betaine possessed potent inhibitory and biofilm eradication properties comparing to all tested products. PHMB is applied as a wound irrigation solution worldwide. However, based on our results, we assume that the PHMB is a promising substance for catheter locking solutions thanks to its safety and high antimicrobial properties.

## Introduction

Central venous catheters (CVCs) are the fastest and most commonly used means of obtaining vascular access in patients requiring hemodialysis. In the United States, they are fitted in close to 80% of persons starting hemodialysis [1]. Depending on the needs and duration of maintenance of vascular access, they can be non-tunnelled temporary catheters or permanent tunnelled catheters with a Dacron cuff, inserted in chronically ill patients or in those waiting for the formation of arteriovenous fistula [2]. Regardless of catheter type, their use carries a high risk of early and late complications. Among late life-threatening complications are catheter-related thrombosis (CRT), central vein stenosis and catheter-related bloodstream infections (CRBSI) [3,4]. The latter is a consequence of earlier colonization of the catheter or the site of its entry by microorganisms, and, like other complications, they lead to more frequent hospitalizations and an increase in patient mortality [5,6]. According to a 2017 report by the European Centre for Disease Prevention and Control, the incidence of catheter infections is estimated to be between 1 and 6.2 cases per 1000 catheter-days [7]. In the publication by Reitzel et al. on parenteral nutrition, this indicator is in the range from 0.35 to 13.64 per 1000 catheter-days [8]. In the studies by Mandolfo et al. on tunnelled catheters, it is lower and amounts to 0.67 per 1000 catheter days [9]. Similar results concerning the occurrence of CRBSI in hemodialysis patients were obtained by Winnicki et al. and Bueloni et al. - 0.67 and 0.79, respectively [10,11]. The lowest indicators are presented in the publication of Van Der Meersch et al., in which they did not exceed 0.24 per 1000 catheter days, regardless of the type of dialysis catheter used [12].

The most common etiological factor of catheter infections is Gram-positive cocci, mainly *Staphylococcus aureus* (31.8%), *Staphylococcus epidermidis* and other coagulase-negative staphylococci (25.3%), as well as *Enterococcus faecalis* (4.9%) [13]. The number of infections caused by multidrug-resistant organisms (MDRO) has also been on the rise [14,15]. Catheter-related infections occur in different locations. The infection can affect the subcutaneous tunnel or the surface around the site of catheter entry. The most severe of them are infections resulting from the presence of microbes in the catheter lumen, leading to bacteremia and the development of a generalized infection. In order to counteract them, standards of practice regarding both safe catheter insertion techniques and professional care for long-term vascular access are periodically updated [16–18]. One crucial measure is the protection of vascular access after a hemodialysis procedure. For this purpose, the inside of the catheter is flushed with saline and then filled with solutions of substances with anticoagulant and antimicrobial properties, which are removed before the next dialysis procedure [19]. Heparin solution was traditionally used to fill dialysis catheters, and in the following years, citrates and taurolidine preparations were introduced into clinical practice. Research on the use of ethanol-containing agents is also underway. Furthermore, antibiotic solutions in combination with anticoagulants (heparin or trisodium citrate) are used in antimicrobial lock therapy (ALT). Nowadays, the most common antibiotics applied are aminoglycosides, beta-lactams, fluoroquinolones or glycopeptides [20,21]. Currently, however, this practice is recommended only for patients at high risk of catheter-related infections (CRBSI >3.5/1000 catheter days, *S*. *aureus* nasal carriers) and is not recommended for routine use due to the very rapidly increasing microbial drug resistance as well as other drug side effects [22]. The topical administration of antibiotics makes it impossible to control their concentration in the catheter lumen, where they can become diluted. Too low concentration of the antibiotic means a lack of its antimicrobial effectiveness. In addition, it leads to the selection of microbes acquiring drug resistance mechanisms, which makes it difficult to choose an effective antibiotic for the treatment of catheter-related bacteremia. The above is confirmed in the results of clinical observations conducted by Landry et al., who used a solution of gentamycin with heparin for catheter locking. In the first six months, they achieved a significant decrease in infections, followed by a growth in the number of bacteremias caused by gentamycin-resistant staphylococci and bacteria of the genus *Enterococcus spp*., which in 4 cases out of 24 led to the patient’s death [23].

Taurolidine is a non-antibiotic agent with proven antibacterial properties used for dialysis catheter locking. Most often used at a concentration of 2%, it can also be combined in a solution, at a concentration of 1.35%, with anticoagulant drugs: heparin, 4% citrate or urokinase [24,25]. Taurolidine has bactericidal and anti-adhesion effects by preventing the formation of biofilm within the catheter and its port [19]. It has a broad spectrum of action, including Gram-positive and Gram-negative organisms, aerobic and anaerobic microbes, as well as bacteria with resistance mechanisms such as MRSA (methicillin-resistant *Staphylococcus aureus*) and VRE (vancomycin-resistant *Enterococcus*) [26]. It also has antifungal activity [27,28]. A solution of trisodium citrate is also used for catheter locking. At a concentration of 4%, it exhibits only anticoagulant activity, as does heparin, while higher concentrations (30% and 46.7%) have an additional antimicrobial effect, involving, among others binding the calcium ions, which are indispensable for bacteria. In 2000, the U.S. Food and Drug Administration (FDA) announced that the use of high concentrations of citrate might cause dangerous complications for the patient, such as cardiac arrest associated with hypocalcaemia. Medical literature includes reports on the use of lower citrate concentrations (7%) in combination with compounds intended to provide antimicrobial activity, such as methylene blue or ethanol [29]. In clinical practice, attempts are made to use other compounds as well. In 2019, El-Hennawy et al. published the results of studies that showed that the use of sodium bicarbonate (NaHCO_3_), effectively reduced the number of catheter infections [30].

As the current agents used do not completely prevent catheter-related bacteremia, the searches for new substances are still ongoing. Polyhexanide (polyhexamethylene biguanide, PHMB) is a compound with antimicrobial properties which in recent years has gained an importance in antiseptics and infection prevention [31–34]. It was shown that polyhexanide-betaine is effective against multi-drug resistant bacteria and their biofilms [35–37]. In terms of structure, polyhexanide resembles antimicrobial peptides and, like them, binds to bacterial cell membranes impairing them and leading to cell lysis. It is also possible that PHMB blocks the DNA replication process [38]. It is characterized by a broad spectrum of antimicrobial action [39]. For increased effectiveness, it is used in combination with betaine—a surfactant compound that facilitates the removal of impurities and additionally disrupts the process of producing signalling molecules used by bacteria in biofilm formation. The efficacy of polyhexanide has been reported in both *in vitro* and *in vivo* studies, where its activity was compared to other compounds used in the prevention of wound and bladder infections in catheterized patients. A reduced number of infections was also observed when this compound was used to prevent exit-site infections of peritoneal catheters [31]. So far, it has not been registered for use as a solution to protect the catheter lumen from microbial colonization.

This paper includes a report on our pilot studies undertaken to evaluate the antimicrobial activity of a solution of polyhexanide-betaine (PHMB-B) and sodium bicarbonate as compared to the most commonly used agentsforcatheter locking. The idea to carry out an *in vitro* study was inspired by promising results bicarbonate use as an effective lock solution (30).

## Materials and methods

### Bacterial strains

The present study used the following reference strains from the American Type Culture Collection (ATCC) belonging to the Chair and Department of Pharmaceutical Microbiology and Parasitology at Wroclaw Medical University: *Staphylococcus aureus* ATCC 33591, *Staphylococcus aureus* ATCC 6538, *Klebsiella pneumoniae* ATCC 4352, *Enterococcus faecium* ATCC 19434, *Enterobacter cloacae* ATCC 13047 and 20 clinical strains isolated from blood and from dialysis catheters. Clinical isolates were obtained from the Academic Center for Laboratory Diagnostics (ACDL), which perform routine analysis for patients hospitalized in the University Hospital in Wroclaw at the departments of anesthesiology and intensive care, neurology, nephrology, general surgery, angiology, cardiology, geriatrics and internal and occupational diseases. Clinical strains are collected at ACDL in University Hospital in Wroclaw constantly during routine microbiological diagnostics and transferred to the Department of Pharmaceutical Microbiology and Parasitology under an agreement between the ACDL and the Department. The tested clinical strains included methicillin-resistant *Staphylococcus aureus* (MRSA; n = 12), methicillin-sensitive *Staphylococcus aureus* (MSSA); n = 3), methicillin-resistant coagulase-negative staphylococci, MRCNS; n = 1), *Enterococcus faecalis* (n = 1) and *Klebsiella pneumoniae* (n = 2) and *Enterobacter cloacae* (n = 1).

### Test substances

Antimicrobial effects of four substances were studied, two of which: 2% taurolidine (Geistlich Pharma, Switzerland) and 30% trisodium citrate (BBraun Avitum, Italy) are registered as dialysis catheter lock solutions. The other compounds were 8.4% sodium bicarbonate (Polpharma, Poland) used in the treatment of metabolic or lactic acidosis (bicarbonate) and suggested as a potential catheter lock solution (30), and 0.1% polyhexanide with the addition of 0.1% betaine in the form of Prontosan (BBraun Medical AG, Switzerland), which hereinafter will be abbreviated as PHMB-B. 0.9% aqueous sodium chloride solution (Chempur, Poland) was used as a control.

### Methods

The antimicrobial activity of the test solutions was assessed by determining the minimum inhibitory concentration (MIC), and the minimum biofilm eradication concentration (MBEC) required to eradicate biofilm from the surface of a polystyrene plate. The ability of strains to form biofilm on the surface of the HemoStar Long-Term Hemodialysis catheter 14.5F (BARD Access Systems, USA) was also tested as well as the degree of biofilm eradication from the catheter surface by the test solutions.

#### Minimum inhibitory concentration (MIC)

To determine the MIC value, 24-hour plankton cultures of the tested strains were grown in tryptic soy broth (TSB, VWR Chemicals, USA), from which suspensions with a density of 1–1.5 x 10^6^ CFU/ml were prepared using a Densilameter® II densimeter (Erba Lachema, Czech Republic). 100 μl of the suspensions were applied to the rows of a 96-well microtitre plate (VWR International, Poland). Independently, serial dilutions of the test substances were prepared in the range from 2 – 1024x in the TSB medium, and 100 μl of each was added to the previously grown bacterial cultures on the plate. The density of the suspension in the wells ranged from 2–8 x 10^5^ CFU/ml. For each strain, a growth control was performed on a TSB medium, and medium sterility control was prepared. The samples were incubated for 24 hours at 37°C and shaken at 400 rpm in Plate Shaker-Thermostat PST-60HL-4 incubator (Biosan, Latvia). After incubation, 20 μl of 1% tetrazolium chloride (TTC, PanReac AppliChem, Germany) was added to each well and incubated for a further 2 hours at 37°C. The results were obtained visually based on the change of colour in the wells with live microbes to red. The test was performed in 3 repetitions for all strains and individual compounds. Due to differences in baseline concentrations of the tested substances (1–300 g/L), MIC values are presented as a percentage concentration (%), taking the baseline concentration of the substances as 100%.

#### Minimum biofilm eradication concentration (MBEC)

To determine the MBEC values of the compounds, 24-hour cultures of the test microorganisms were grown on microtitration plates in 100 μl TSB. The initial suspension density in the well was 2–8 x 10^5^ CFU/ml. The plates were incubated for 24 hours at 37°C under static conditions. After incubation, the medium was removed from the biofilm using a Reglo Digital peristaltic pump (Ismatec, Germany). 200 μl of previously prepared serial dilutions of the test substances were added to the wells (dilution range 0 – 512x). 200 μl of TSB was added to the growth control wells and to the sterility control well. The plates thus prepared were incubated for 24h at 37°C under static conditions. After incubation, the medium was removed from the biofilm, and 200 μl of 0.1% TTC solution in TSB was added to each well and incubated for 2 hours at 37°C. The test results were obtained visually, like during the determination of the MIC value. The test was performed in 3 repetitions for all strains and for individual compounds.

#### Biofilm forming ability on the catheter surface

Tests to assess biofilm formation ability on catheters were conducted using a polyurethane HemoStar Long-Term Hemodialysis Catheter 14.5 F (BARD Access Systems, USA). The microbes’ ability to form biofilm was assessed using a modified Richards method. This method utilizes the metabolic potential of bacterial cells in reducing a tetrazolium salt to a red formazan product. The test compared the metabolic activity of bacteria in the biofilm formed on the surface of the tested dialysis catheter. 5 mm long segments of the catheter were placed in the wells of a 24-well plate and covered with 2 ml of bacterial suspension with a density of 2–8 x 10^5^ CFU/ml. The plates were incubated for 24 hours at 37°C under static conditions. After incubation, the catheter segments were transferred to new 24-well plates, and 2 ml of 0.1% TTC solution in TSB was added. The samples were incubated for 2 hours at 37°C. Then the catheter segments were transferred to new sterile wells and covered with 2 ml of 96% ethanol (Chempur, Poland). The plates were incubated for 30 min at 37°C and shaken at 400 rpm. The stained solution was transferred to a 96-well plate, and the colour intensity was measured at a wavelength of λ = 490 nm in the Multiscan® GO spectrophotometer (Thermo Scientific, USA). 96% ethanol solution was used as a negative control.

#### Evaluation of antimicrobial activity against biofilm formed on the catheter surface

The clinical strains that, during the assessment of biofilm-forming capacity (Fig 3), showed the highest deviation from the mean absorbance value (0.1563) were used in the subsequent phase of the study. The rate of eradication by the test substances of the biofilm from the catheter surface was assessed in relation to the biofilm of reference strains MRSA ATCC 33591, MSSA ATCC 6538, EClo ATCC 13047, EFm ATCC 19434, KP ATCC 4352 and selected clinical strains whose biofilm-forming ability was strong or poor. Strains for which absorbance values were below the 10th percentile (0.0548) were labelled as "poor biofilm-formers" and included MRSA11 and EFs1. The absorbance of strains that were "strong biofilm-formers" (MRSA12 and MRSA8) was higher than the 90th percentile (0.3349). The strains’ biofilm culture was grown according to the procedure described in materials and methods. After incubation, the catheter segments were transferred to sterile wells of 48-well plates, and 1 ml of the tested solutions was added to them at baseline concentrations. 0.9% NaCl solution served as growth control. The samples were incubated for 24 hours at 37°C under static conditions. Catheter segments were then transferred to clean 24-well plates and stained with 2 ml of 0.1% TTC in TSB. The samples were incubated for 2 hours at 37°C. After the set time, the catheters were placed in clean wells of 24-well plates and covered with 2 ml of 96% ethanol. The entire sample was shaken for 30 min at 37°C at 400 rpm. The colour intensity was measured spectrophotometrically at wavelength λ = 490 nm in four repetitions for each test. The rate of biofilm eradication from the catheter surface [ER] was calculated using the formula:

$$Eradication\;rate\;[\%]=100\%-\frac{AbsK}{AbsB*100\%}$$

where AbsK corresponds to the mean absorbance of the growth control and AbsB to the mean absorbance obtained after the effects of the test substances on the biofilm. The experiment was performed three times for all strains and all studied compounds.

### Statistical analysis

The dot plots of MIC and MBEC values were made in Microsoft Excel, Microsoft 365, https://office.microsoft.com/excel. The statistical analysis was made using GraphPad Prism version 8.0.1 for Windows, GraphPad Software, San Diego, California USA, www.graphpad.com. Normal distribution was evaluated by the Shapiro-Wilk test with a significance level of α = 0.05. The effects of the test substances on biofilm formed on catheter segments were compared using the non-parametric Kruskal-Wallis rank ANOVA with post-hoc Dunn’s test with a significance level of α = 0.05. Results for which the probability value p < 0.05 were considered statistically significant.

### Ethics statement

All tests performed in this research and described in the article was an *in vitro* study and did not require patients participation. All bacterial strains used for this study are the part of Department of Pharmaceutical Microbiology and Parasitology collection at Wroclaw Medical University. Clinical strains are constantly collected at Academic Center for Laboratory Diagnostics (ACDL) in University Hospital in Wroclaw during routine microbiological diagnostics and transferred to the Department of Pharmaceutical Microbiology and Parasitology under an agreement between the Hospital and the Department. Blood and dialysis catheters were not collected from patients for scientific research purposes but only for microbiology diagnostics of infection.

## Results

### Determination of the minimum inhibitory concentration (MIC)

**Fig 1:**
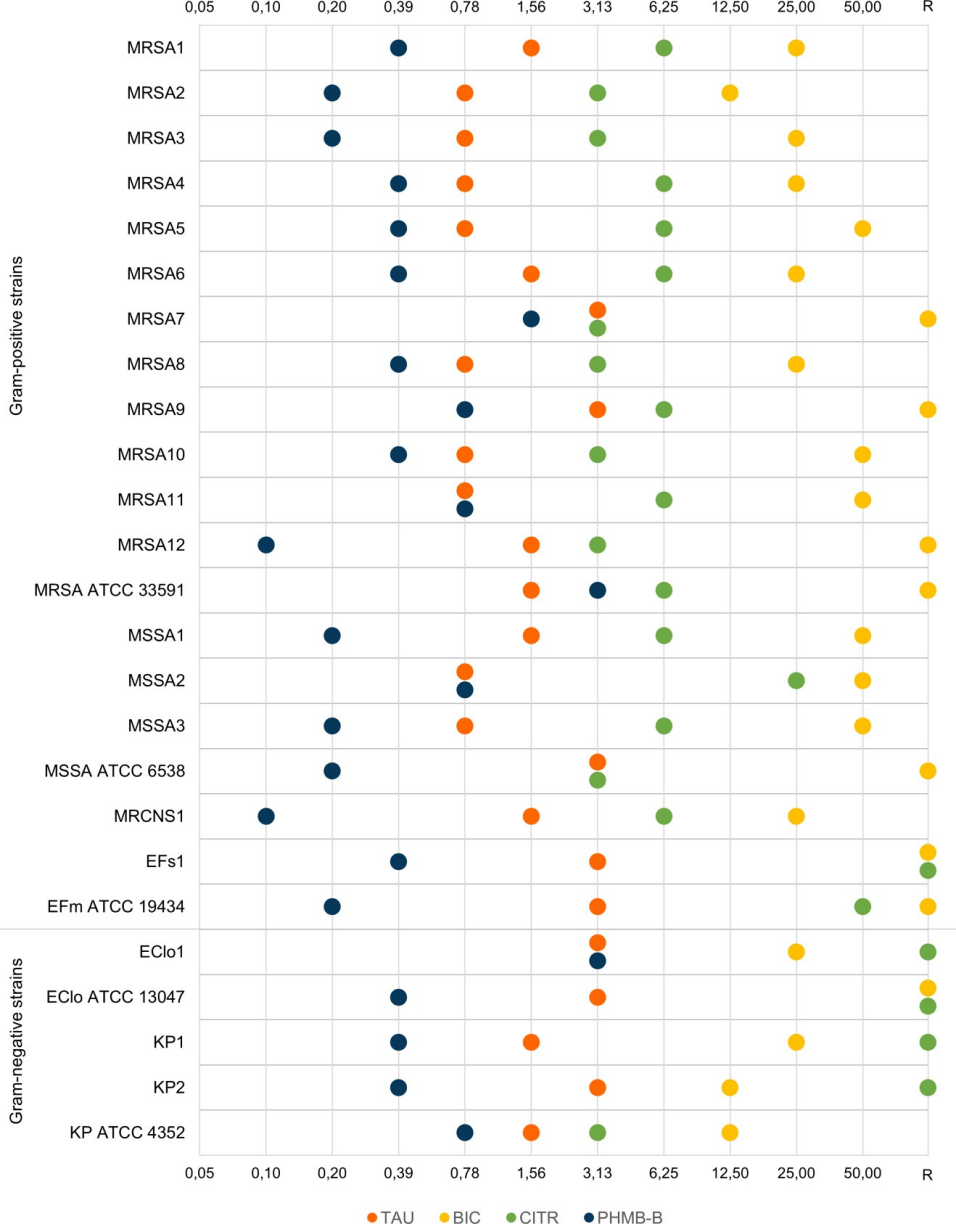
Minimum concentrations of preparations inhibiting the growth of bacteria (MIC). TAU–taurolidine, BIC–bicarbonate, CITR- trisodium citrate, PHMB-B–polyhexanide-betaine, R–no activity in the test concentration range, MRSA–methicillin-resistant *Staphylococcus aureus*, MSSA–methicillin-sensitive *Staphylococcus aureus*, MRCNS–methicillin-resistant coagulase-negative *Staphylococcus hominis*, EFs–*Enterococcus faecalis*, EFm–*Enterococcus faecium*, Eclo–*Enterobacter cloacae*, KP–*Klebsiella pneumoniae*, ATCC–American Type Culture Collection.

The antibacterial activity of the 4 analyzed substances was tested on 25 strains of bacteria belonging to 6 different species. The results of MIC determinations of the tested solutions for each bacterial strain are shown in Fig 1. Full data are available in Supplementary Materials in S1 Table.

After serial dilution of the compounds at in-use concentrations, only taurolidine and polyhexanide-betaine showed antimicrobial activity against all tested strains (Fig 1). Taurolidine MIC values ranged from 0.78% to 3.125% of its baseline concentration, i.e. 156 mg/L to 625 mg/L, respectively. Thus, this substance inhibited the growth of bacteria after 32-fold to 128-fold dilution. The highest sensitivity to taurolidine (MIC 0.78% of baseline concentration, 0.156 g/L) was demonstrated by 9 strains of *S*. *aureus* (MRSA2-5, MRSA8, MRSA10-11; MSSA2-3). Twice higher MIC values were determined for five strains of *S*. *aureus* (MRSA1, MRSA6, MRSA12, MRSA ATCC 33591, MSSA1), *S*. *hominis* (MRCNS1), the clinical and the reference strain of *K*. *pneumoniae* (KP1, *K*. *pneumoniae ATCC* 4352). Three strains of *S*. *aureus* (MRSA7, MRSA9 and MSSA ATCC 6538), *Enterococcus* strains (EF1 and *E*. *faecium* ATCC 19434) and *E*.*cloacae* strains (EClo1, EClo ATCC 13047) and the clinical strain *K*.*pneumoniae* (KP2) had the highest MIC value (3.125%; 625 mg/L) (Fig 1).

The MIC values for PHMB-B ranged from 0.098% to 3.125% of the baseline concentration of the solution, i.e. 0.98 mg/L– 31.25 mg/L, respectively. The substance inhibited bacterial growth when diluted 32–1024 fold. For 18 strains, the percentage of MIC of PHMB-B was lower than the lowest MIC value [%] for taurolidine. PHMB-B at a concentration of 0.098%, (0.98 mg/L) inhibited the growth of clinical strains MRSA12 and MRCNS1. A concentration of 1.95 mg/L (MIC 0.195%) effectively inhibited the growth of five strains of *S*. *aureus* (MRSA2-3, MSSA1, MSSA3 and MSSA ATCC 6538) and of the *E*. *faecium* ATCC 19434 strain. For 10 strains, including 6 strains of *S*. *aureus* (MRSA1, MRSA4-6, MRSA8, MRSA10), clinical strain *E*. *faecalis* (EFs1), 2 strains of *K*. *pneumoniae* (KP1, KP2) and reference strain *E*. *cloacae* ATCC 13047, the MIC value was 3.91 mg/L (0.391%). A MIC of 7.81 mg/L (0.78%) was recorded for three clinical strains of *Staphylococcus aureus* (MRSA9, MRSA11 and MSSA2) and for the reference strain *K*. *pneumoniae* ATCC 4352. Only the growth of MRSA7 was inhibited at a higher concentration of 15.63 mg/L (1.56%). The highest MIC value (31.25 mg/L, 3.125%) was determined for the clinical strain *E*. *cloacae* (EClo1) and for the MRSA reference strain ATCC 33591.

The compound with trisodium citrate contained 300 g/L of the active substance (30%). This was the highest concentration of an active substance in the compared formulations. Despite such a high concentration of the active substance, for 5 out of 25 test strains, the MIC value was > 50% of baseline concentration (Fig 1). These were 2 Gram-negative *E*. *cloacae* and 2 clinical strains of *K*. *pneumoniae*. The clinical strain *Enterococcus faecalis* was also resistant to citrate within the tested concentration range. The lowest MIC values (9.375 g/L, 3.125% and 18.75 g/L; 6.25%) were determined for 16 *S*. *aureus* strains, both MRSA and MSSA (MRSA2-3,7–8,10,12 and MRSA 1,4–6, 9,11, ATCC 33591, MSSA 1,3 respectively) and also for *S*. *hominis* (MRCNS1) and reference strain *K*. *pneumoniae* ATCC 4352. For the clinical strain MSSA2 the MIC value was 75 g/L (25%). The concentration of trisodium citrate inhibiting the growth of the *E*. *faecium* ATCC 19434 reference strain was 150 g/L (50%). Clinical strains of K. *pneumoniae*, *E*. *cloacae*, *E*. *faecalis* and the *E*. *cloacae* ATCC 13047 reference strain did not show sensitivity to the compound diluted in the medium. They were resistant within the selected concentration range (Fig 1).

The lowest antimicrobial activity was recorded for sodium bicarbonate (MIC range 12.5 - >50% of baseline concentration, 10.5 - >42 g/L), for which the MIC value for nearly 30% of the strains was higher than 50% of the baseline concentration of this formulation. Bicarbonate inhibited the growth of 17 out of 25 tested strains. The lowest MIC was 10.5 g/L (12.5%), and it was observed for two clinical strains (MRSA2 and KP2) and for the reference strain *K*. *pneumoniae* ATCC 4352. A concentration of 21 g/L (25%) of the test substance represented the MIC value for five clinical strains of MRSA (MRSA1, MRSA3-4, MRSA6 and MRSA8) and *S*. *hominis* (MRCNS1), *E*. *cloacae* (EClo1) and one *K*. *pneumoniae* (KP1). The growth of clinical MSSA strains (MSSA1-3) and three MRSA strains (MRSA5, MRSA10-11) was inhibited by a concentration of 42 g/L (50%) of sodium bicarbonate. The remaining tested strains showed resistance to bicarbonate within the tested concentration range.

### Determination of the minimum biofilm eradication concentration (MBEC)

**Fig 2:**
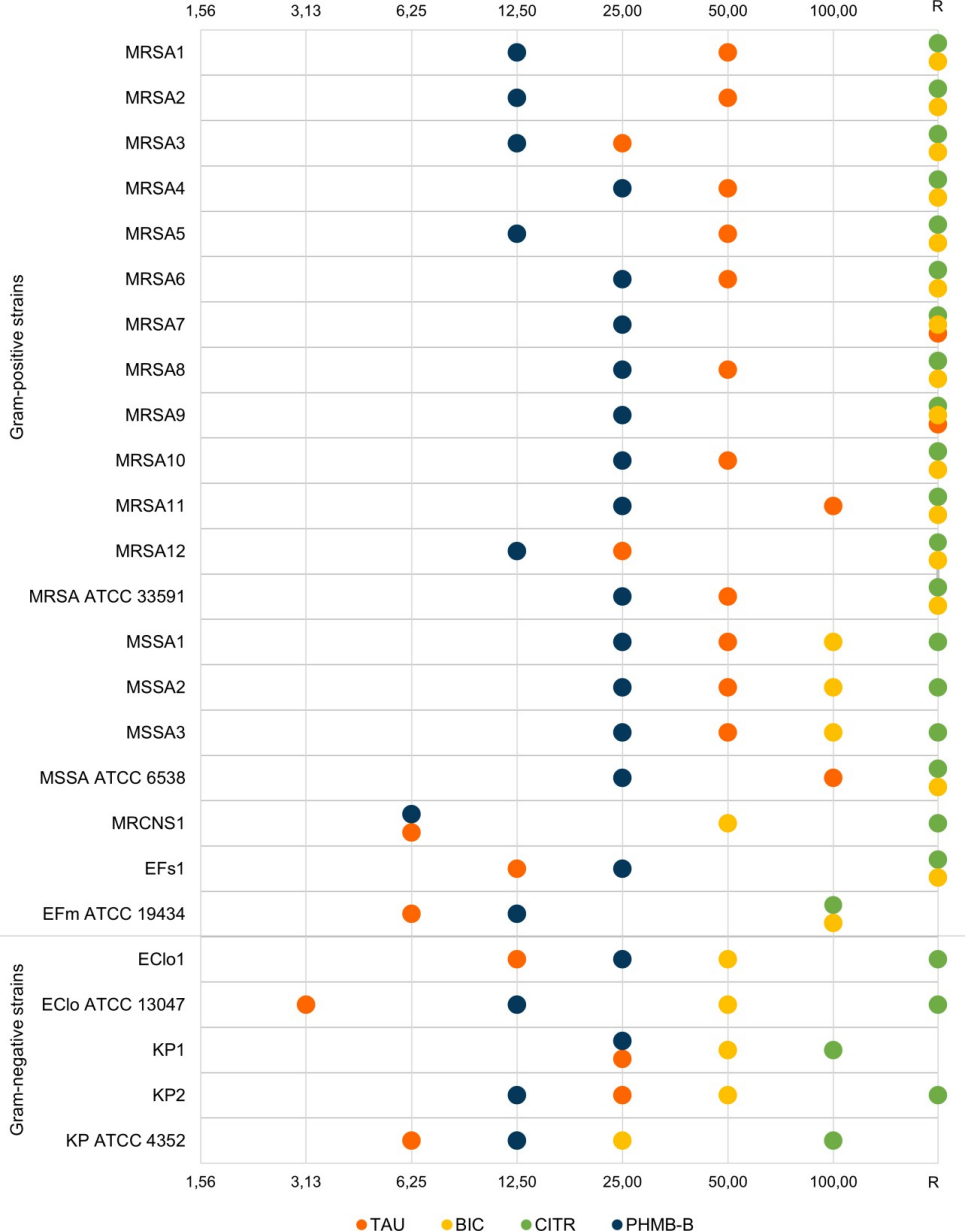
Minimum concentrations [%] of the compounds able to eradicate biofilm (MBEC). TAU–taurolidine, BIC–bicarbonate, CITR—trisodium citrate, PHMB-B–polyhexanide-betaine, R–no activity within the tested concentration range, MRSA–methicillin-resistant *Staphylococcus aureus*, MSSA–methicillin-sensitive *Staphylococcus aureus*, MRCNS–methicillin-resistant coagulase-negative *Staphylococcus hominis*, EFs–*Enterococcus faecalis*, EFm–*Enterococcus faecium*, EClo–*Enterobacter cloacae*, KP–*Klebsiella pneumoniae*, ATCC–American Type Culture Collection.

Once the MIC value was established, the next step of the study involved the determination of the minimum concentrations of the tested substances able to eradicate biofilm from the surface of the polystyrene plate. The results of the determinations of MBEC values of the tested solutions for individual bacterial strains are shown in Fig 2. Full data are available in Supplementary Materials in S2 Table.

The highest effect on bacterial cells in biofilm cultures on polystyrene plates was observed for PHMB-B. The MBEC values of this solution for all the strains, regardless of species, ranged from 6.25% to 25% of baseline concentration (0.063 g/L to 0.25 g/L, respectively. The lowest MBEC was determined for the clinical strain *S*. *hominis* (MRCNS1), against which PHMB-B was active after 16-fold dilution. For five clinical strains of *S*. *aureus* (MRSA1-3, MRSA5 and MRSA12), the clinical strain *K*. *pneumoniae* (KP2) and reference strains: *K*. *pneumoniae* ATCC 4352, *E*. *cloacae* ATCC 13047, *E*. *faecium* ATCC 19434, the MBEC was 0.125 g/L (12.5%). The biofilm of the remaining strains was eradicated in a 4-fold dilution of the tested compound (0.25 g/L, 25%).

Taurolidine also demonstrated the ability to eradicate biofilm. However, MBEC values differed within the tested strain group. The lowest MBEC of 0.625 g/L (3.125%) was determined for the reference strain *E*. *cloacae* ATCC 13047. MBEC twice as high was observed for the *S*. *hominis* strain (MRCNS1), and the reference strains *E*. *faecium* ATCC 19434 and *K*. *pneumoniae* ATCC 4352. The MBEC for the clinical strains *E*. *faecalis* (EFs1) and *E*. *cloacae* (EClo1) was 2.5 g/L (12.5%). Two MRSA strains (MRSA3 and MRSA12) and two *K*. *pneumoniae* strains (KP1, KP2) had an MBEC of 5 g/L (25%). For 11 *S*. *aureus* strains (MRSA1-2, MRSA4-6, MRSA8, MRSA10, MRSA ATCC 33591, MSSA1-3), MBEC was 10 g/L (50%). By contrast, MBEC of 20 g/L (100%), i.e. an undiluted formulation with taurolidine, eradicated the biofilm of two strains of *Staphylococcus aureus* (MRSA11 and MSSA ATCC 6538). The other two clinical strains of *S*. *aureus* (MRSA7 and MRSA9) were resistant to taurolidine at a concentration of 20 g/L.

Bicarbonate eradicated biofilm less effectively than the above-discussed compounds. The MBEC value within the tested concentration range was determined for only 10 of the 25 strains tested. The lowest value of MBEC (21 g/L, 25%) was determined for the reference strain *K*. *pneumoniae* ATCC 4352. Bicarbonate at a concentration of 42 g/L (50%) was able to eradicate the biofilm of four strains of Gram-negative bacteria (*E*. *cloacae*, clinical strains of *K*. *pneumoniae*) and one Gram-positive bacteria strain–*S*. *hominis* (MRCNS1). The above results indicate that the use of bicarbonate eradicated the biofilm of strains isolated from patients only at a concentration of 50% and higher. The activity of the undiluted compound (84 g/L, 100%) was limited to the biofilm of methicillin-sensitive clinical strains of *S*. *aureus* (MSSA1-3) and the reference strain *E*. *faecium* ATCC 19434. The biofilm structure of the clinical strain *E*. *faecalis* (EFs1) and of all tested methicillin-resistant *S*. *aureus* strains (MRSA1-12, MRSA ATCC 33591) and of the reference strain MSSA ATCC 6538 was characterized by resistance to the baseline concentration of sodium bicarbonate.

The lowest antibiofilm efficacy was demonstrated by a 30% solution of trisodium citrate. Biofilm eradication at baseline concentrations was observed only for 3 strains: *K*. *pneumoniae* (KP1 and *K*. *pneumoniae* ATCC 4352) and *E*. *faecium* ATCC 19434. Biofilm created by 22 of the 25 strains tested was resistant to the 30% solution of trisodium citrate.

### Determination of biofilm-forming ability on the catheter surface

**Fig 3:**
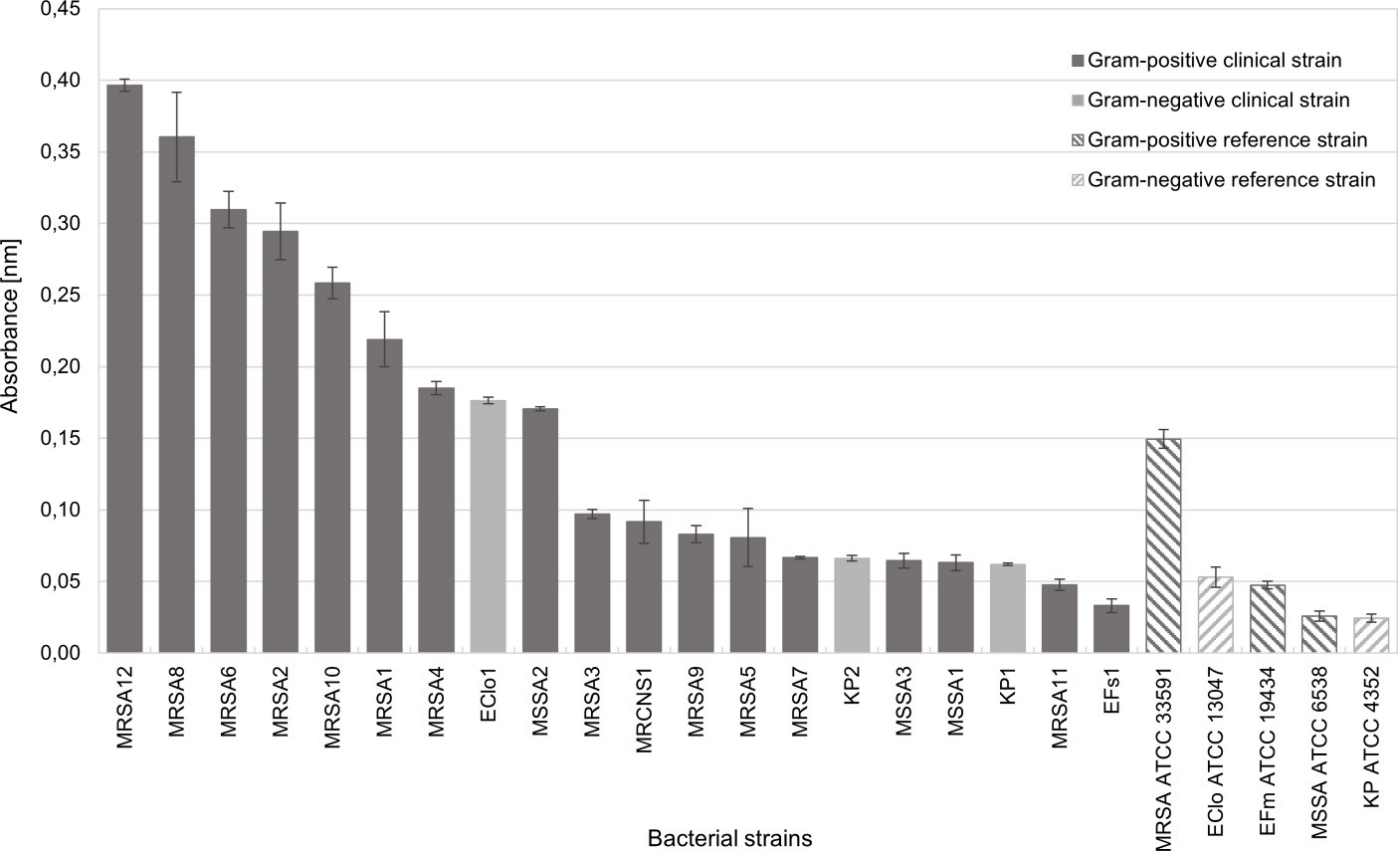
Biofilm forming ability on the surface of a polyurethane catheter. MRSA–methicillin-resistant *Staphylococcus aureus*, MSSA—methicillin-sensitive *Staphylococcus aureus*, MRCNS—methicillin-resistant coagulase-negative *Staphylococcus hominis*, EFs–*Enterococcus faecalis*, EFm–*Enterococcus faecium*, EClo–*Enterobacter cloacae*, KP–*Klebsiella pneumoniae*, ATCC–American Type Culture Collection.

In order to compare the obtained results with the activity of the tested solutions against the biofilm formed on dialysis catheters, the ability to form biofilm on polyurethane catheters was first tested for all strains. Seven of the tested clinical strains showed metabolic activity in biofilm twice that of the corresponding species of reference strains (Fig 3). Full data are available in Supplementary Materials in S3 Table.

Only for the clinical strains of the genus *Enterococcus sp*. (EFs1) and 5 MRSA strains (MRSA3, MRSA5, MRSA7, MRSA9 and MRSA11), the reference strains displayed higher activity. The highest metabolic activity was observed for the biofilm of methicillin-resistant strains of *S*. *aureus*, followed by MRSA12, MRSA8, MRSA6, MRSA2 and MRSA10. Among Gram-negative bacteria, the highest metabolic activity was observed in the biofilm of clinical strains EClo1 and KP1-2 and the reference strain of *E*. *cloacae* ATCC 13047. The biofilm of *E*. *faecalis* (EFs1), MRSA11, MSSA ATCC 6538 and *K*. *pneumoniae* ATCC 4352 showed the lowest metabolic activity.

### Antimicrobial activity against biofilm formed on the catheter surface

**Fig 4:**
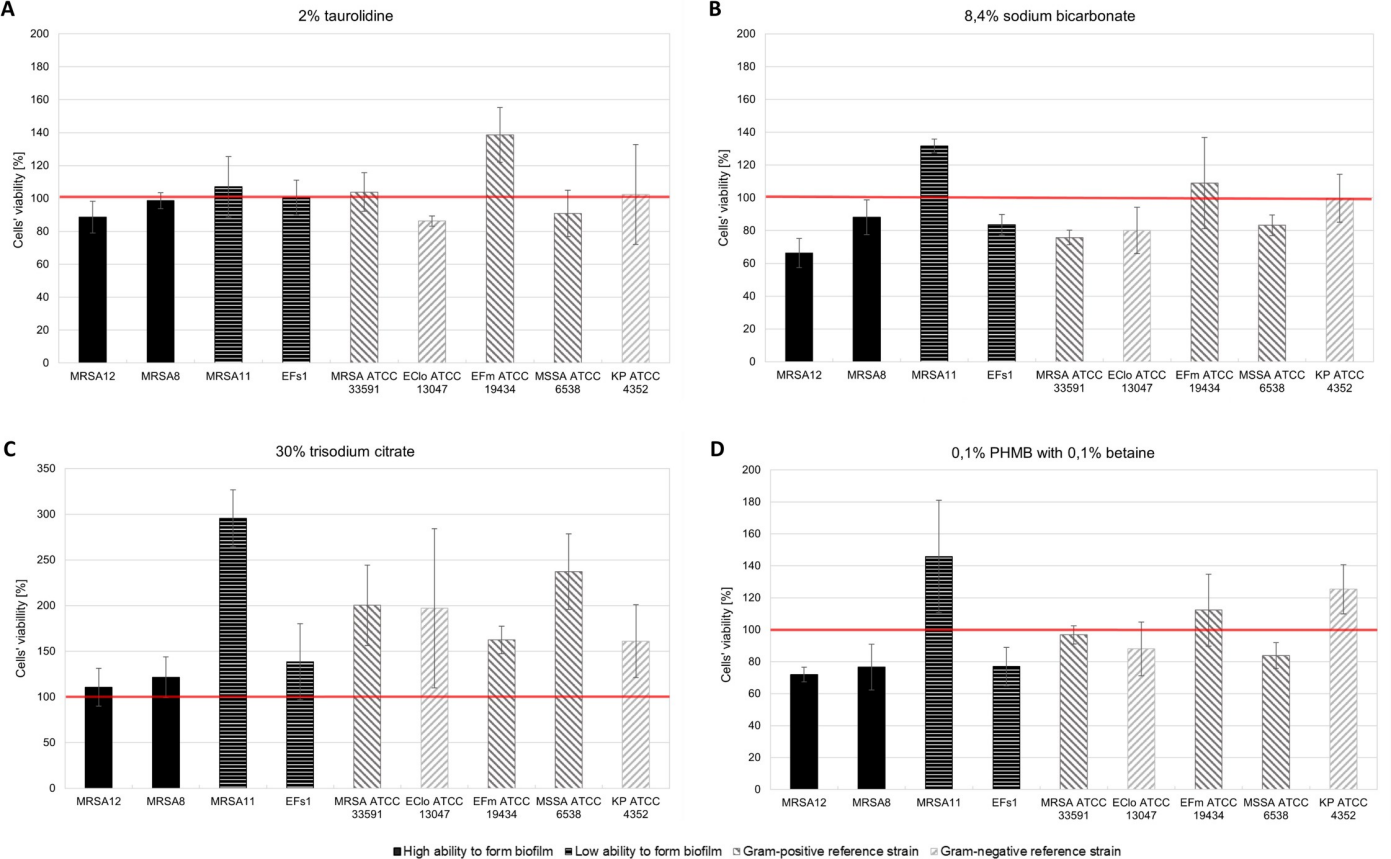
Anti-biofilm activity of compounds against biofilm formed on the dialysis catheter. (a) 2% taurolidine, (b) 8.4% sodium bicarbonate (c) 30% trisodium citrate, (d) 0.1% polyhexanide with 0.1% betaine (PHMB-B). The red line shows the metabolic activity of the bacteria in control [%]; MRSA–methicillin-resistant *Staphylococcus aureus*, MSSA–methicillin-sensitive *Staphylococcus aureus*, EFs–*Enterococcus faecalis*, EFm–*Enterococcus faecium*, EClo–*Enterobacter cloacae*, KP–*Klebsiella pneumoniae*, ATCC–American Type Culture Collection.

The assessment of the rate of biofilm eradication from the surface of a dialysis catheter by baseline concentrations of the tested substances was carried out for the selected strains with strong and weak metabolic activity in the biofilm. Reference strains *S*. *aureus* ATCC 33591 and ATCC 6538, *E*. *faecium* ATCC 19434, *K*. *pneumoniae* ATCC 4352, *E*. *cloacae* ATCC 13047 and clinical strains MRSA12, MRSA8 (strong biofilm producers), MRSA11 and EFs1 (poor biofilm producers) were selected for the experiment. A 24-hour biofilm culture incubated in 0.9% NaCl solution was used as growth control. The results of the experiment are shown in Fig 4. Full data are available in Supplementary Materials in S4 Table.

2% taurolidine solution reduced the number of metabolically active cells in the biofilm in 4/9 of the tested strains. The rate of eradication was the highest for *E*. *cloacae* ATCC 13047 (ER = 13.71%, SD = 3.06), MRSA12 (ER = 11.35%, SD = 9.73) and MSSA ATCC 6538 (ER = 9.11%, SD = 14.01) and the lowest for MRSA8 (ER = 1.37%, SD = 4.78). Bicarbonate at a baseline concentration of 8.4% showed activity against 7/9 of the tested strains. A decrease in the number of metabolically active cells was observed in MRSA12 (ER = 33.66%, SD = 8.84), MRSA8 (ER = 11.85%, SD = 10.54), MRSA ATCC 33591 (ER = 24.22%, SD = 4.47), *E*. *cloacae* ATCC 13047 (ER = 19.95%, SD = 14.14), EFs1 (ER = 16.48%, SD = 6.24), MSSA ATCC 6538 (ER = 16.76%, SD = 6.28). The use of 0.1% solution of polyhexanide-betaine reduced the number of metabolically active cells in 6/9 of the tested strains: MRSA12 (ER = 28.12%, SD = 4.53), MRSA8 (ER = 23.40%, SD = 14.32), EFs1 (ER = 23.07%, SD = 12.2), MSSA ATCC 6538 (ER = 16.17%, SD = 8.06), *E*. *cloacae* ATCC 13047 (ER = 11.97%, SD = 16.9), MRSA ATCC 33591 (ER = 3.21%, SD = 5.71). 30% citrate was the only tested compound that did not show any biofilm eradication ability against any of the tested strains. Taurolidine, bicarbonate and polyhexanide-betaine demonstrated activity against the clinical isolates of methicillin-resistant S*taphylococcus aureus* (MRSA12, MRSA8—strong biofilm producers), as well as against reference strains MSSA ATCC 6538 and *E*. *cloacae* ATCC 13047. PHMB-B and sodium bicarbonate had an inhibitory effect on the clinical strain EFs1 and the MRSA ATCC 33591 reference strain. Additionally, bicarbonate was the only tested substance to demonstrate activity against the biofilm of the reference strain *K*. *pneumoniae* ATCC 4352. None of the compounds showed activity against the clinical strain MRSA11 and the reference strain *E*. *faecium* ATCC 19434.

The results of the statistical analysis of the efficacy of the tested compounds on biofilm are presented in Table 1.

**Table 1:** Comparison of the compounds’ efficacy against biofilm formed on the catheter surface.

Pair compared^a^	Occurrence of statistical significance	Level of statistical significance^b^
TAU vs. GC	No	ns
BIC vs. GC	No	ns
CITR vs. GC	Yes	*
PHMB-B vs. GC	No	ns
TAU vs. BIC	No	ns
TAU vs. CITR	Yes	***
TAU vs. PHMB-B	No	ns
BIC vs. CITR	Yes	***
BIC vs. PHMB-B	No	ns
PHMB-B vs. CITR	Yes	***

^a^ TAU–taurolidine, BIC–sodium bicarbonate, CITR–trisodium citrate, PHMB-B–polyhexanide-betaine, GC–growth control

^b^ ns–no statistical significance, p value: * (.033), ** (.002), *** (< .001).

Based on the assessment of metabolic activity of microbes in the biofilm treated with baseline concentrations of the compounds, the only partial ability of three substances to eradicate the biofilm of selected bacterial strains was demonstrated. They were taurolidine, polyhexanide-betaine and bicarbonate, among which no statistically significant differences were found. None of the substances showed a broad spectrum of action, simultaneously against Gram-positive and Gram-negative organisms. 30% solution of trisodium citrate showed no effect on the biofilm formed by the tested strains, and its activity differed statistically significantly from the remaining compounds.

## Discussion

From the studies carried out and from the analysis of the obtained results, it follows that the tested compounds have a diversified antimicrobial activity on planktonic bacterial cultures. Polyhexanide-betaine and taurolidine (Fig 1) showed the highest activity. Inhibition of bacterial growth regardless of species was observed not only at the highest concentration of these compounds (50%) but also after dilution 32–128x (taurolidine) and 32–1024x (PHMB-B). Therefore, it can be assumed that the use of the above solutions at baseline concentrations can prevent catheter colonization by both Gram-negative bacilli and Gram-positive cocci. However, it should not be concluded that these formulations may be diluted before administration in order to reduce the cost of their use. They may undergo further dilution in the catheter lumen. The above is suggested by studies conducted by Polaschegg, which concluded that when administering a catheter lock, up to 20% of its volume can enter the circulatory system, thereby reducing its concentration in the catheter lumen [40,41]. Similarly, Doorenbos et al. pointed out that the administered preparations become diluted when mixed with the liquid in the catheter [42]. After administration and maintaining within the catheter, even at 32-fold dilution, taurolidine should still preserve its antimicrobial efficacy against the species most commonly colonizing catheters. Polyhexanide-betaine, although there is currently no preparation, including it registered for hemodialysis catheters locking, has comparable activity to taurolidine, especially against *Staphylococcus aureus* (Fig 1). The statistical tests carried out did not reveal any significant differences between the MIC values of the two substances for the above group of pathogens (p>0.05). At the same time, polyhexanide-betaine is active against different microbial species [31]. It can therefore be a potential new catheter locking solution, although (like taurolidine) it does not have any anticoagulant effects. In turn, trisodium citrate, which has such activity, showed an antibacterial effect against S*taphylococcus spp* strains comparable with taurolidine, even at 16-fold dilution (Fig 1). However, its activity was weaker against other species, including Gram-negative bacilli. An antimicrobial effect weaker than that of citrate was observed for sodium bicarbonate (Fig 1). It inhibited the growth of certain strains after 2–8 fold dilution. It should be emphasized that, unlike trisodium citrate, it most effectively inhibited the growth of Gram-negative *K*. *pneumoniae* bacilli. Thus, the obtained results confirm the effectiveness of baseline concentrations of compounds in preventing the colonization of catheter lumen only for 2% taurolidine and for polyhexanide-betaine, which has not yet been used as a catheter lock solution. The above is especially true if their effect is compared to the use of the only saline, heparin or 4% citrate, which is devoid of any antimicrobial action. When assessing the efficacy of the tested substances, the values of their minimum inhibitory concentrations should be considered. Clinical studies published so far on the use of taurolidine as a catheter clock solution also confirm its effectiveness in reducing CRBSI [43–46]. Taurolidine concentration of 2% present in commercially available preparations exceeds several times the MIC50 value determined for different Gram-positive and Gram-negative bacteria (<512 mg/L). The MIC values obtained in this study are similar and range from 156 mg/L to 625 mg/L. It should also be pointed out that the obtained effects of the solution depend on the frequency of its application [44]. On the basis of data published so far, it can be concluded that combining this substance in solutions with citrate or heparin to improve the thrombolytic effect of the solution does not reduce its antimicrobial activity [47,48].

Another important property of the tested solutions, which was the subject of the present study was their ability to destroy the bacterial cells making up biofilm. Biofilm is a structure that, owing to the presence of substances secreted by the microorganisms forming an extracellular matrix, is much more difficult to penetrate, not only by cells and molecules of the immune system but also by antibiotics and other antimicrobial agents [49]. Numerous *in vitro* studies show that biofilms have a higher tolerance to the tested substances than planktonic cultures [50–52]. This phenomenon was also observed in the studies carried out for the purposes of this paper, and the obtained minimum biofilm eradication concentrations (MBEC) were several times higher than the MIC values (Fig 2). Polyhexanide-betaine proved to be the most effective in the eradication of biofilm formed on polystyrene plates. It remained active against all microbial species studied even at a 4-fold dilution. 2% taurolidine solution inhibited metabolic activity in the biofilm in more than 80% of the studied strains after at least 2-fold dilution. For two methicillin-resistant *Staphylococcus aureus* strains with different biofilm-producing abilities (MRSA7 and MRSA9), taurolidine showed no effect and was active against MRSA11 and MSSA ATCC 6538 at baseline concentration only. No antibiofilm effects were observed in 15 strains after the application of sodium bicarbonate and in 22 strains incubated in the presence of trisodium citrate. The latter solution was active at baseline concentration only against *K*. *pneumoniae* ATCC 4352 and KP1 strains, as well as against *E*. *faecium* reference strains. An important observation is the repeated higher activity of bicarbonate against Gram-negative bacilli compared to its activity against Gram-positive bacteria.

On the other hand, no proof was obtained for the tested compounds to have a significant effect on the biofilm formed on the surface of a polyurethane catheter as compared to the control of 0.9% NaCl solution (Fig 4). The tested substances caused only a slight reduction in the metabolic activity of cells in the biofilm in individual strains vs. the control (Fig 4). In addition, it can be concluded that trisodium citrate not only did not show any biofilm eradication activity but increased the survival of the cells present in it. These results are in line with those obtained by Shanks et al., who demonstrated that citrate and heparin could stimulate biofilm formation by *S*. *aureus* [53]. On the other hand, Olthof et al. cite studies that have shown that *Klebsiella pneumoniae* can metabolize trisodium citrate by fermentation [27].

It should be stressed that the research methodology adopted here has some limitations. When TTC is used to assess biofilm eradication, bacterial survival is determined based on their metabolic activity rather than changes in the number of living cells. Therefore, it is possible that a single spectrophotometric measurement may indicate the survival of numerous cells with low TTC metabolism or of a few cells that show high metabolic activity. Similar conclusions can be drawn from the results of studies presented in the paper by Haney et al. [54]. *S*. *aureus* strains with high biofilm biomass were at the same time characterized by weak growth. It is now known that the presence in the biofilm of metabolically inactive cells depends on the growth phase of the microbial population. Additionally, given that bacteria in the biofilm incubated in the presence of baseline concentrations of compounds during 24 hours had limited access to nutrients, they could have slowed down their metabolism by entering a stationary growth phase, and only after having been transferred to the culture medium with TTC, could have reactivated it [55,56]. This could explain the weak effect of taurolidine, which for its antimicrobial activity, requires detachment of three free methyl groups as a result of the activity of bacterial enzymes. The detached methyl groups bind to murein components in the bacterial cell wall [44,57]. Similarly, the chelation of calcium ions by trisodium citrate and sodium bicarbonate may have been of limited significance to slow-metabolizing cells. On the other hand, there are reports that microbes such as *S*. *aureus*, *S*. *epidermidis* and *P*. *aeruginosa* can survive up to 7 days in a solution of 8.4% sodium bicarbonate [58].

The results obtained may indicate that polyhexanide-betaine may be worth considering in the prevention of catheter colonization although it is not yet registered as a lock solution. Its use would require wider clinical trials regarding cytotoxicity and safety and assessment of its biocompatibility with the plastics from which catheters are made. Finally, it has to be pointed that strict adherence to hygienic precautions in the dialysis unit is the most important issue to decrease the number of CRBSI [59].

## Conclusions

The results obtained may indicate the high significance of taurolidine and polyhexanide-betaine in the prevention of catheter colonization. The effect of sodium bicarbonate was not satisfactory. It is worth noting, that the use of antiseptics and other bactericidal agents may be of limited significance in the event that the biofilm structure has already developed within the catheter. What may be important here is not only the efficacy with which the agents are able to kill bacterial cells but also the fact that the biofilm present in the catheter containing dead microbes can facilitate recolonization of the catheter by other microbial species. It is therefore crucial to use preventive measures, including strict compliance with all catheter care procedures to prevent microbial colonization. These activities include antimicrobial lock therapy with the use of substances that effectively inhibit the growth of microorganisms and biofilm formation.

## Supporting information

S1 Table Minimal Inhibitory Concentrations (MIC) of tested compounds presented in g/L and % of initial substance concentration. Strains: MRSA1-12 methicillin-resistant Staphylococcus aureus, MSSA1-3 methicillin-susceptible Staphylococcus aureus, MRCNS1 Staphylococcus hominis, KP1-2 Klebsiella pneumoniae, EFs1 Enterococcus faecalis, EClo1 Enterobacter cloacae, EFm Enterococucs faecium; TAU–taurolidine, BIC–bicarbonate, CITR–trisodium citrate, PHMB-B–polyhexanide-betaine; ATCC American Type Culture Collection; R—strain resistant to the initial substance concentration.
(DOCX)

S2 Table Minimal biofilm eradication concentrations (MBEC) of tested substances presented in g/L and % of initial substance concentration. Clinical strains: MRSA1-12 methicillin-resistant Staphylococcus aureus, MSSA1-3 methicillin-susceptible Staphylococcus aureus, MRCNS1 Staphylococcus hominis, KP1-2 Klebsiella pneumoniae, EF1 Enterococcus faecalis, EClo1 Enterobacter cloacae, EFm Enterococcus faecium; TAU–taurolidine, BIC–bicarbonate, CITR–trisodium citrate, PHMB-B–polyhexanide-betaine; ATCC American Type Culture Collection; R—strain resistant to the starting substance concentration.
(DOCX)

S3 Table Ability to form biofilm on the catheter surface. Presented as absorbance values of six replicates (REP1-6), also average (AVG) and standard deviation (SD) are calculated for tested type and clinical strains: MRSA1-12 methicillin-resistant *Staphylococcus aureus*, MSSA1-3 methicillin-susceptible *Staphylococcus aureus*, MRCNS1 Staphylococcus *hominis*, KP1-2 *Klebsiella pneumoniae*, EFs1 *Enterococcus faecalis*, EClo1 *Enterobacter cloacae*, EFm *Enterococcus faecium*, ATCC American Type Culture Collection.
(DOCX)

S4 Table Eradication of biofilm formed on the catheter surface of tested strains. Presented as average (AVG) and standard deviations (SD) of three replications as a percentage of survived cells in comparison to the growth control; MRSA1-12 methicillin-resistant *Staphylococcus aureus*, MSSA1-3 methicillin-susceptible *Staphylococcus aureus*, MRCNS1 *Staphylococcus hominis*, KP1-2 *Klebsiella pneumoniae*, EF1 *Enterococcus faecalis*, EClo1 *Enterobacter cloacae*, EFm *Enterococcus faecium;* ATCC–American Type Culture Collection; TAU–taurolidine, BIC–bicarbonate, CITR–trisodium citrate, PHMB-B–polyhexanide-betaine.
(DOCX)
