# Supplementary material for: Antimicrobial activity of hemodialysis catheter lock solutions in relation to other compounds with antiseptic properties
Source: PLoS One. 2021 Oct 7;16(10):e0258148. doi: 10.1371/journal.pone.0258148 (PMC8496847; doi:10.1371/journal.pone.0258148)
Supplement: S1 Table — Strains: MRSA1-12 methicillin-resistant Staphylococcus aureus, MSSA1-3 methicillin-susceptible Staphylococcus aureus, MRCNS1 Staphylococcus hominis, KP1-2 Klebsiella pneumoniae, EFs1 Enterococcus faecalis, EClo1 Enterobacter cloacae, EFm Enterococucs faecium; TAU–taurolidine, BIC–bicarbonate, CITR–trisodium citrate, PHMB-B–polyhexanide-betaine; ATCC American Type Culture Collection; R—strain resistant to the initial substance concentration. (DOCX) [file pone.0258148.s001.docx]

**S1 Table. Minimal Inhibitory Concentrations (MIC) of tested compounds presented in g/L and % of initial substance concentration.** Strains: MRSA1-12 methicillin-resistant Staphylococcus aureus, MSSA1-3 methicillin-susceptible Staphylococcus aureus, MRCNS1 Staphylococcus hominis, KP1-2 Klebsiella pneumoniae, EFs1 Enterococcus faecalis, EClo1 Enterobacter cloacae, EFm Enterococucs faecium; TAU – taurolidine, BIC – bicarbonate, CITR – trisodium citrate, PHMB-B – polyhexanide-betaine; ATCC American Type Culture Collection; R - strain resistant to the initial substance concentration.

| **STRAIN** | **TAU** | | **BIC** | | **CITR** | | **PHMB-B** | |
| --- | --- | --- | --- | --- | --- | --- | --- | --- |
|  | **g/L** | **%** | **g/L** | **%** | **g/L** | **%** | **g/L** | **%** |
| **MRSA1** | 0,313 | 1,560 | 21,0 | 25,0 | 18,750 | 6,250 | 0,004 | 0,391 |
| **MRSA2** | 0,156 | 0,780 | 10,5 | 12,5 | 9,375 | 3,125 | 0,002 | 0,195 |
| **MRSA3** | 0,156 | 0,780 | 21,0 | 25,0 | 9,375 | 3,125 | 0,002 | 0,195 |
| **MRSA4** | 0,156 | 0,780 | 42,0 | 50,0 | 9,375 | 3,125 | 0,004 | 0,391 |
| **MRSA5** | 0,156 | 0,780 | 21,0 | 25,0 | 18,750 | 6,250 | 0,004 | 0,391 |
| **MRSA6** | 0,156 | 0,780 | 42,0 | 50,0 | 18,750 | 6,250 | 0,004 | 0,391 |
| **MRSA7** | 0,313 | 1,560 | 21,0 | 25,0 | 18,750 | 6,250 | 0,004 | 0,391 |
| **MRSA8** | 0,156 | 0,780 | 21,0 | 25,0 | 9,375 | 3,125 | 0,004 | 0,391 |
| **MRSA9** | 0,625 | 3,125 | R | R | 18,750 | 6,250 | 0,008 | 0,780 |
| **MRSA10** | 0,625 | 3,125 | R | R | 9,375 | 3,125 | 0,016 | 1,560 |
| **MRSA11** | 0,156 | 0,780 | 42,0 | 50,0 | 18,750 | 6,250 | 0,008 | 0,780 |
| **MRSA12** | 0,313 | 1,560 | R | R | 9,375 | 3,125 | 0,001 | 0,098 |
| **MSSA1** | 0,313 | 1,560 | 42,0 | 50,0 | 18,750 | 6,250 | 0,002 | 0,195 |
| **MSSA2** | 0,156 | 0,780 | 42,0 | 50,0 | 75,000 | 25,000 | 0,008 | 0,780 |
| **MSSA3** | 0,156 | 0,780 | 42,0 | 50,0 | 18,750 | 6,250 | 0,002 | 0,195 |
| **MRCNS1** | 0,313 | 1,560 | 21,0 | 25,0 | 18,750 | 6,250 | 0,001 | 0,098 |
| **KP1** | 0,313 | 1,560 | 21,0 | 25,0 | R | R | 0,004 | 0,391 |
| **KP2** | 0,625 | 3,125 | 10,5 | 12,5 | R | R | 0,004 | 0,391 |
| **EFs1** | 0,625 | 3,125 | R | R | R | R | 0,004 | 0,391 |
| **EClo1** | 0,625 | 3,125 | 21,0 | 25,0 | R | R | 0,031 | 3,125 |
| **MRSA ATTC 33591** | 0,313 | 1,560 | R | R | 18,750 | 6,250 | 0,031 | 3,125 |
| **MSSA ATTC 6538** | 0,625 | 3,125 | R | R | 9,375 | 3,125 | 0,002 | 0,195 |
| **EC ATCC 25922** | 0,625 | 3,125 | 10,5 | 12,5 | 150,0 | 50,0 | 0,004 | 0,391 |
| **KP ATTC 4352** | 0,313 | 1,560 | 10,5 | 12,5 | 75,0 | 25,0 | 0,008 | 0,780 |
| **EFm ATTC 19434** | 0,625 | 3,125 | R | R | 150,0 | 50,0 | 0,002 | 0,195 |
| **EClo ATTC 13047** | 0,625 | 3,125 | R | R | R | R | 0,004 | 0,391 |
