# Supplementary material for: Antimicrobial activity of hemodialysis catheter lock solutions in relation to other compounds with antiseptic properties
Source: PLoS One. 2021 Oct 7;16(10):e0258148. doi: 10.1371/journal.pone.0258148 (PMC8496847; doi:10.1371/journal.pone.0258148)
Supplement: S3 Table — Presented as absorbance values of six replicates (REP1-6), also average (AVG) and standard deviation (SD) are calculated for tested type and clinical strains: MRSA1-12 methicillin-resistant Staphylococcus aureus, MSSA1-3 methicillin-susceptible Staphylococcus aureus, MRCNS1 Staphylococcus hominis, KP1-2 Klebsiella pneumoniae, EFs1 Enterococcus faecalis, EClo1 Enterobacter cloacae, EFm Enterococcus faecium, ATCC American Type Culture Collection. (DOCX) [file pone.0258148.s003.docx]

**S3 Table. Ability to form biofilm on the catheter surface.** Presented as absorbance values of six replicates (REP1-6), also average (AVG) and standard deviation (SD) are calculated for tested type and clinical strains: MRSA1-12 methicillin-resistant *Staphylococcus aureus*, MSSA1-3 methicillin-susceptible *Staphylococcus aureus*, MRCNS1 Staphylococcus *hominis*, KP1-2 *Klebsiella pneumoniae*, EFs1 *Enterococcus faecalis*, EClo1 *Enterobacter cloacae,* EFm *Enterococcus faecium,* ATCC American Type Culture Collection*.*

| **STRAIN** | **REP1** | **REP2** | **REP3** | **REP4** | **REP5** | **REP6** | **AVG** | **SD** |
| --- | --- | --- | --- | --- | --- | --- | --- | --- |
| **MRSA1** | 0,191 | 0,256 | 0,217 | 0,219 | 0,214 | 0,217 | 0,219 | 0,019 |
| **MRSA2** | 0,263 | 0,286 | 0,294 | 0,297 | 0,330 | 0,297 | 0,295 | 0,020 |
| **MRSA3** | 0,090 | 0,097 | 0,099 | 0,099 | 0,099 | 0,099 | 0,097 | 0,003 |
| **MRSA4** | 0,175 | 0,185 | 0,189 | 0,188 | 0,186 | 0,187 | 0,185 | 0,005 |
| **MRSA5** | 0,125 | 0,064 | 0,075 | 0,074 | 0,072 | 0,072 | 0,081 | 0,020 |
| **MRSA6** | 0,299 | 0,289 | 0,312 | 0,313 | 0,317 | 0,329 | 0,310 | 0,013 |
| **MRSA7** | 0,068 | 0,068 | 0,066 | 0,066 | 0,066 | 0,067 | 0,067 | 0,001 |
| **MRSA8** | 0,326 | 0,367 | 0,338 | 0,397 | 0,404 | 0,331 | 0,360 | 0,031 |
| **MRSA9** | 0,087 | 0,077 | 0,094 | 0,082 | 0,079 | 0,078 | 0,083 | 0,006 |
| **MRSA10** | 0,235 | 0,261 | 0,259 | 0,262 | 0,267 | 0,266 | 0,258 | 0,011 |
| **MRSA11** | 0,050 | 0,047 | 0,046 | 0,056 | 0,044 | 0,045 | 0,048 | 0,004 |
| **MRSA12** | 0,389 | 0,398 | 0,401 | 0,400 | 0,399 | 0,392 | 0,397 | 0,004 |
| **MSSA1** | 0,074 | 0,060 | 0,062 | 0,066 | 0,059 | 0,057 | 0,063 | 0,005 |
| **MSSA2** | 0,171 | 0,171 | 0,173 | 0,168 | 0,169 | 0,171 | 0,171 | 0,002 |
| **MSSA3** | 0,075 | 0,065 | 0,063 | 0,061 | 0,060 | 0,064 | 0,064 | 0,005 |
| **MRCNS1** | 0,079 | 0,113 | 0,092 | 0,103 | 0,095 | 0,068 | 0,092 | 0,015 |
| **KP1** | 0,061 | 0,063 | 0,063 | 0,061 | 0,062 | 0,063 | 0,062 | 0,001 |
| **KP2** | 0,064 | 0,064 | 0,068 | 0,066 | 0,065 | 0,069 | 0,066 | 0,002 |
| **EFs1** | 0,032 | 0,031 | 0,032 | 0,044 | 0,030 | 0,031 | 0,033 | 0,005 |
| **EClo1** | 0,174 | 0,176 | 0,175 | 0,174 | 0,177 | 0,181 | 0,176 | 0,002 |
| **MRSA ATTC 33591** | 0,150 | 0,163 | 0,143 | 0,146 | 0,148 | 0,146 | 0,149 | 0,007 |
| **MSSA ATTC 6538** | 0,029 | 0,026 | 0,026 | 0,031 | 0,022 | 0,021 | 0,026 | 0,003 |
| **EC ATCC 25922** | 0,135 | 0,138 | 0,122 | 0,125 | 0,127 | 0,130 | 0,129 | 0,006 |
| **KP ATTC 4352** | 0,023 | 0,023 | 0,031 | 0,024 | 0,022 | 0,024 | 0,024 | 0,003 |
| **EFm ATTC 19434** | 0,044 | 0,046 | 0,053 | 0,049 | 0,046 | 0,047 | 0,047 | 0,003 |
| **EClo ATTC 13047** | 0,049 | 0,053 | 0,053 | 0,044 | 0,052 | 0,067 | 0,053 | 0,007 |
