# Supplementary material for: Antimicrobial activity of hemodialysis catheter lock solutions in relation to other compounds with antiseptic properties
Source: PLoS One. 2021 Oct 7;16(10):e0258148. doi: 10.1371/journal.pone.0258148 (PMC8496847; doi:10.1371/journal.pone.0258148)
Supplement: S4 Table — Presented as average (AVG) and standard deviations (SD) of three replications as a percentage of survived cells in comparison to the growth control; MRSA1-12 methicillin-resistant Staphylococcus aureus, MSSA1-3 methicillin-susceptible Staphylococcus aureus, MRCNS1 Staphylococcus hominis, KP1-2 Klebsiella pneumoniae, EF1 Enterococcus faecalis, EClo1 Enterobacter cloacae, EFm Enterococcus faecium; ATCC–American Type Culture Collection; TAU–taurolidine, BIC–bicarbonate, CITR–trisodium citrate, PHMB-B–polyhexanide-betaine. (DOCX) [file pone.0258148.s004.docx]

**Table S4. Eradication of biofilm formed on the catheter surface of tested strains.** Presented as average (AVG) and standard deviations (SD) of three replications as a percentage of survived cells in comparison to the growth control; MRSA1-12 methicillin-resistant *Staphylococcus aureus*, MSSA1-3 methicillin-susceptible *Staphylococcus* *aureus*, MRCNS1 *Staphylococcus* *hominis*, KP1-2 *Klebsiella* *pneumoniae*, EF1 *Enterococcus* *faecalis*, EClo1 *Enterobacter* *cloacae*, EFm *Enterococcus* *faecium;* ATCC – American Type Culture Collection; TAU – taurolidine, BIC – bicarbonate, CITR *–* trisodium citrate, PHMB-B – polyhexanide-betaine.

| **ABILITY TO FORM BIOFILM** | **STRAIN** | **TAU** | | **BIC** | | **CITR** | | **PHMB+B** | |
| --- | --- | --- | --- | --- | --- | --- | --- | --- | --- |
|  |  | **AVG** | **SD** | **AVG** | **SD** | **AVG** | **SD** | **AVG** | **SD** |
| HIGH | **MRSA8** | 98,627 | 4,786 | 88,151 | 10,539 | 121,488 | 22,187 | 76,603 | 14,327 |
| LOW | **MRSA11** | 107,053 | 18,436 | 131,581 | 4,289 | 295,492 | 31,175 | 145,876 | 35,061 |
| HIGH | **MRSA12** | 88,646 | 9,731 | 66,335 | 8,843 | 110,615 | 20,757 | 71,877 | 4,532 |
| LOW | **EFs1** | 100,592 | 10,474 | 83,524 | 6,242 | 138,529 | 41,724 | 76,928 | 12,025 |
| HIGH | **MRSA ATCC 33591** | 103,814 | 11,731 | 75,784 | 4,474 | 200,305 | 44,135 | 96,788 | 5,707 |
| LOW | **MSSA ATCC 6538** | 90,894 | 14,015 | 83,244 | 6,286 | 237,149 | 41,272 | 83,830 | 8,062 |
| HIGH | **EClo ATCC 13047** | 86,294 | 3,061 | 80,048 | 14,145 | 197,190 | 87,142 | 88,031 | 16,903 |
| LOW | **EFm ATCC 19434** | 138,608 | 16,618 | 109,010 | 27,849 | 162,492 | 15,071 | 112,263 | 22,550 |
| HIGH | **KP ATCC 4352** | 102,404 | 30,382 | 99,725 | 14,531 | 161,116 | 39,950 | 125,377 | 15,349 |
